# Publisher’s Notices

**Published:** 1868-05-15

**Authors:** 


### PUBLISHER'S NOTICES.

---

THE MAILS.—Great pains have been taken to have the JOURNAL regularly and promptly sent to each subscriber. Those who fail to receive it regularly will confer a favor by advising us of the fact, and those who have failed to receive any of the back numbers, can secure them by addressing this office.

BACK NUMBERS.—A very few numbers of the JOURNAL, for January 15, 1868, are needed at this office. For all those received before June 15, we will allow the sum of twenty-five cents each on account.

TO CORRESPONDENTS.—Daily letters are received at the office of the JOURNAL, asking for information with regard to various subjects for the writer. Will those who address us in this manner, asking for a written reply, remember to inclose a stamp for postage? Hereafter, we can not undertake to reply to those who do not comply with this regulation.

During the coming month those of our subscribers who have not received a statement of the amount of their indebtedness to the JOURNAL, will have been so notified — some of them for the second time. We can not again notify those who are delinquent. Will not those subscribers who have suffered the back dues to accumulate, make a strenuous effort and square their accounts on our books? The JOURNAL has undergone a heavy expense in changing form and style. Other improvements are being projected, which, completed, will place it in the foremost rank of the medical publications of the day. We have resolved to supply the profession in the West with a periodical whose pages shall reflect the progress that is constantly taking place in the science and its practice. These improvements all cost money, and it is imperatively necessary for those who are in debt to us to pay up promptly. Without the support and encouragement of our subscribers, we can not successfully conduct a publication to which we give our time and our efforts to the end that it may be interesting and instructive to them. We are certain that having said thus much, our readers will feel it both a duty and pleasure to respond with promptness to this, our last call.

